# Gβγ-mediated activation of protein kinase D exhibits subunit specificity and requires Gβγ-responsive phospholipase Cβ isoforms

**DOI:** 10.1186/1478-811X-11-22

**Published:** 2013-04-05

**Authors:** Winnie WI Lau, Anthony SL Chan, Lydia SW Poon, Jing Zhu, Yung H Wong

**Affiliations:** 1 Division of Life Science and the Biotechnology Research Institute, Hong Kong University of Science and Technology, Clear Water Bay, Kowloon, Hong Kong; 2 State Key Laboratory of Molecular Neuroscience, Hong Kong University of Science and Technology, Clear Water Bay, Kowloon, Hong Kong

**Keywords:** G proteins, Gα subunits, Gβγ dimers, PLCβ, PKD

## Abstract

**Background:**

Protein kinase D (PKD) constitutes a novel family of serine/threonine protein kinases implicated in fundamental biological activities including cell proliferation, survival, migration, and immune responses. Activation of PKD in these cellular activities has been linked to many extracellular signals acting through antigen receptor engagement, receptor tyrosine kinases, as well as G protein-coupled receptors. In the latter case, it is generally believed that the Gα subunits of the G_q_ family are highly effective in mediating PKD activation, whereas little is known with regard to the ability of Gβγ dimers and other Gα subunits to stimulate PKD. It has been suggested that the interaction between Gβγ and the PH domain of PKD, or the Gβγ-induced PLCβ/PKC activity is critical for the induction of PKD activation. However, the relative contribution of these two apparently independent events to Gβγ-mediated PKD activation has yet to be addressed.

**Results:**

In this report, we demonstrate that among various members in the four G protein families, only the Gα subunits of the G_q_ family effectively activate all the three PKD isoforms (PKD1/2/3), while Gα subunits of other G protein families (G_s_, G_i_, and G_12_) are ineffective. Though the Gα subunits of G_i_ family are unable to stimulate PKD, receptors linked to G_i_ proteins are capable of triggering PKD activation in cell lines endogenously expressing (HeLa cells and Jurkat T-cells) or exogenously transfected with (HEK293 cells) Gβγ-sensitive PLCβ_2/3_ isoforms. This indicates that the G_i_-mediated PKD activation is dependent on the released Gβγ dimers upon stimulation. Further investigation on individual Gβγ combinations (i.e. Gβ_1_ with Gγ_1–13_) revealed that, even if they can stimulate the PLCβ activity in a comparable manner, only those Gβ_1_γ dimers with γ_2_, γ_3_, γ_4_, γ_5_, γ_7_, and γ_10_ can serve as effective activators of PKD. We also demonstrated that G_i_-mediated PKD activation is essential for the SDF-1α-induced chemotaxis on Jurkat T-cells.

**Conclusions:**

Our current report illustrates that Gβγ dimers from the G_i_ proteins may activate PKD in a PLCβ_2/3_-dependent manner, and the specific identities of Gγ components within Gβγ dimers may determine this stimulatory action.

## Background

Protein kinase D (PKD) constitutes a novel family of diacylglycerol (DAG)-responsive serine/threonine protein kinases with different structural, enzymological and regulatory properties from the protein kinase C (PKC) family members [1-3]. To date, three members of the PKD family have been identified: human PKD1 (its mouse ortholog being PKCμ), and the more recently identified PKD2 and PKD3 (also named PKCν), among which PKD1 is the most extensively characterized isoform. Emerging studies have revealed that PKDs are implicated in a complex array of fundamental biological activities, including cell survival [4], migration [5], proliferation [6], and immune responses [7]. In addition, growing evidence links PKDs to signal transduction pathways in tumor development and cancer progression. In many cases, specific PKD isoforms are dysregulated in cancer cells [8].

All PKDs share a common modular structure, with a tandem repeat of zinc finger-like cysteine-rich motifs at their NH_2_ termini that display high affinity for DAG or phorbol ester, a pleckstrin homology domain (PH domain) for negative regulation of kinase activity [9], and a C-terminal catalytic domain containing transphosphorylation and autophosphorylation sites. Activation of PKD isoforms is generally attributed to phosphorylation at a pair of highly conserved serine residues (for human: Ser^738^ and Ser^742^ in PKD1; Ser^706^ and Ser^710^ in PKD2; Ser^731^ and Ser^735^ in PKD3) in the activation loop of the kinase domain by PKC. As PKC can be activated by many extracellular signals, stimulation of PKD isoforms has been demonstrated by antigen receptor engagement [10], stimulation of receptor tyrosine kinases (RTKs) such as platelet-derived growth factors (PDGF) receptors [11] and vascular endothelial growth factor (VEGF) receptors [12], as well as activation of various G protein-coupled receptors (GPCRs). Among the large GPCR family, receptors with preferential coupling to G_q_, including those responsive to bombesin, vasopressin, endothelin, bradykinin [13], cholecystokinin [14], tachykinin [15] and angiotensin II have been demonstrated to activate PKD in a variety of cell types. Other G protein members like G_12_ and G_13_ have also been proposed to activate PKD3 in a PKC- and Rac-dependent manner [16]. In addition, it has been reported that G_q_, G_i_ and G_12/13_ may cooperate in LPA-induced PKD activation [17], but the relative contribution of specific G protein subunits (e.g. Gα_i_ versus Gβγ) to PKD activation remains undefined.

The functional specificity of G proteins was originally accredited to the Gα subunits, with the Gβγ dimers being viewed as negative regulators of G protein signaling. Yet, there is growing evidence that Gβγ dimers can also act as active mediators in signal transduction, thus conferring an additional level of signal specificity [18-20]. The Gβ identity in the Gβγ dimer imparts selectivity on its interaction with effectors like phospholipase Cβ [21], as well as in the regulation of neutrophil function [22]. Moreover, since the Gγ component is structurally and expression-wise diverse, it imposes additional complexity in signal transduction. For instance, only certain Gβγ combinations (mainly those containing γ_2_, γ_4_, γ_7_ or γ_9_) are linked to significant STAT3 activation [23]. Functional selectivity of Gγ subunits has also been reported [24-26]; deletion of the *Gng3* gene leads to increased susceptibility to seizures in mice with significant reductions in Gβ_2_ and Gα_i3_ subunit levels in certain brain regions [25], whereas knock-out of the *Gng7* gene is associated with reductions in the Gα_olf_ subunit content and adenylyl cyclase activity of the murine striatum [24]. These observations demonstrate that members of the Gγ subunit family are not functionally interchangeable.

It has been suggested that the interaction between Gβγ and the PH domain of PKD [27], or the Gβγ-induced PLCβ/PKC activity is critical for the induction of PKD activation [28].However, the relative contribution of these two apparently independent events to Gβγ-mediated PKD activation has yet to be addressed. Recently, Gβγ combinations containing Gγ_2_ (i.e. Gβ_1_γ_2_ and Gβ_3_γ_2_) have been shown to be effective activators for PKD [29], but the relevant capabilities of other Gβγ dimers remain unclear.

In this report, we demonstrated that all family members of the G_q_ subfamily (G_q_, G_11_, G_14_, and G_16_) can induce PKD1, PKD2 and PKD3 activation. G_s_ cannot elicit a PKD response, whereas G_i_ members may induce PKD activation in a Gβγ-dependent manner. For the Gβγ-induced PKD activation, even in the presence of PLCβ_2_ or PLCβ_3_, only certain Gβγ dimer combinations are capable of activating the kinase effectively. Moreover, we showed that this selective Gβγ dimer-mediated PKD activation is accompanied by enhanced interaction between the two components when PLCβ_2/3_ is present.

## Materials and methods

### Materials

HEK293 and Jurkat T-cells were obtained from American Type Culture Collection (Rockville, MD). Pertussis toxin (PTX) was purchased from List Biological Laboratories (Campbell, CA). Cell culture reagents including Dulbecco’s phosphate-buffered saline (PBS), trypsin, fetal bovine serum (FBS), penicillin-streptomycin mixture, RPMI 1640 medium, minimum essential medium (MEM), Dulbecco’s modified Eagle’s medium (DMEM) and Lipofectamine PLUS™ were obtained from Invitrogen (Carlsbad, CA). The cDNAs encoding PLCβ_1_, PLCβ_2_ and PLCβ_3_ were obtained from Dr. Richard Ye (University of Illinois at Chicago). Flag-tagged human Gβ_1_ and Gβ_2_, HA-tagged human Gγ_1_, Gγ_2_, Gγ_3_, Gγ_4_, Gγ_5_, Gγ_7_, Gγ_8_, Gγ_9_, Gγ_10_, Gγ_11_, Gγ_12_ and Gγ_13_ cDNA constructs were obtained from UMR cDNA Resource Center (Rolla, MO). Antiserum including anti-Flag and anti-HA were purchased from Roche Molecular Biochemicals (Indianapolis, IN). Cell culture reagents including Lipofectamine Plus™ were obtained from Invitrogen (Carlsbad, CA). Myo-[^3^H] inositol was purchased from DuPont NEN (Boston, MA). M2 affinity gels and protein A-agarose were obtained from Sigma (St. Louis, MO). HA-PKD1 and FLAG-PKD2 constructs were gifts from Dr. J. Van Lint (Katholieke Universiteit Leuven, Belgium), and Myc-PKD3 constructs were kindly provided by Dr. Q. J. Wang (University of Pittsburgh, PA).

### Cell culture and transfection

HEK293 cells were cultured in MEM supplemented with 10% (v/v) FBS, 50 units/ml penicillin, and 50 μg/ml streptomycin. Jurkat T-cells were cultured in RPMI1640 containing 10% (v/v) FBS. For PLC assays and co-immunoprecipitation assays, HEK293 cells were seeded at 60% confluency into 12-well plates or 6-well plates, respectively. Transfection was performed on the following day using Lipofectamine PLUS™ reagents. For the establishment of stable cell lines (293/BK_2_R, 293/β_2_AR and 293/fMLPR), exponentially growing HEK293 cells were transfected with cDNA of BK_2_R, β_2_AR or fMLPR in pcDNA3.1-zeo using Lipofectamine PLUS™. The cells were then selected with Zeocin (50 μg/mL). 293/fMLPR-Gα_16_ cells were established by transient transfection of 293/fMLPR stable cell lines with Gα_16_ in pcDNA3.

### In vitro PKD Assay

Twenty-four hours after transfection, HEK293 cells were serum-starved overnight and then treated with 500 μl (per well) of ice-cold detergent-containing lysis buffer (50 mM Tris–HCl, pH 7.5, 100 mM NaCl, 5 mM EDTA, 40 mM Na_4_P_2_O_7_, 1% Triton X-100, 1 mM dithiothreitol, 200 μM Na_3_VO_4_, 100 μM phenylmethylsulfonyl fluoride, 2 μg/ml leupeptin, 4 μg/ml aprotinin, and 0.7 μg/ml pepstatin). Lysates obtained were subjected to *in vitro* PKD kinase assay. Fifty μl of each supernatant was used for the detection of PKD isoform expression and stimulatory phosphorylation, and the remaining lysate (450 μl) was incubated overnight at 4°C with specific affinity gels to immune-precipitate the corresponding PKD isoform (anti-HA for HA-PKD1; anti-FLAG for FLAG-PKD2; and anti-Myc for Myc-PKD3). The resulting immunoprecipitates were washed twice with lysis buffer and twice with kinase assay buffer (30 mM Tris–HCl, pH 7.4, 10 mM MgCl, and 1 mM DTT). Washed immunoprecipitates were resuspended in 40 μl of kinase assay buffer containing 2.5 mg/ml of Syntide-2 (PLARTLSVAGLPGKK), and the kinase reactions were initiated by the addition of 10 μl of ATP buffer containing 1 μCi of [γ-^32^P]-ATP per sample. After 10-min incubation at 30°C with occasional shaking, the reactions were terminated by adding 100 μl of 75 mM H_3_PO_4_ and spotting 75 μl of the reaction mix onto P-81 phosphocellulose paper. Free [γ-^32^P]-ATP was separated from the labelled substrate by washing the P-81 paper four times (5 min each) in 75 mM H_3_PO_4_. The papers were dried and the radioactivity incorporated into Syntide-2 was determined by scintillation counting.

### Electroporation

The knock down of PKD1, PKD2 and PKD3 was performed by introducing the corresponding PKD isoform-specific siRNA from Invitrogen (Carlsbad, CA, USA) using Nucleofector® Kit V from Lonza (Basel, Switzerland). Briefly, 1×10^6^ cells per sample were resuspended in Nucleofector® Solution and supplement provided at room temperature. siRNA against PKD1, PKD2 or PKD3 (200 pmol each) was added to the samples and then electroporated using the Nucleofector®. Electroporated cells were then incubated at room temperature for 10 min before transferring them into the 12-well plate with culture medium. The knock down of PLCβ_1_, PLCβ_2_ and PLCβ_3_ was performed in similar manner, with the corresponding isoform-specific siRNA obtained from Santa Cruz Biotechnology (Santa Cruz, CA, USA).

### Western blotting analysis

Cells in 12-well plate were lysed in 300 μl of ice-cold lysis buffer (50 mM Tris–HCl, pH 7.5, 100 mM NaCl, 5 mM EDTA, 40 mM NaP_2_O_7_, 1% Triton X-100, 1 mM dithiothreitol, 200 μM Na_3_VO_4_, 100 μM phenylmethylsulfonyl fluoride, 2 μg/ml leupeptin, 4 μg/ml aprotinin and 0.7 μg/ml pepstatin). Clarified lysates were resolved on 1 μ2% SDS-polyacrylamide gels and then transferred to nitrocellulose membranes (Westborough, MA). Stimulatory phosphorylation of PKD1, PKD2, ERK and CREB were detected by their corresponding antisera and horseradish peroxidase-conjugated secondary antisera. The immunoblots were visualized by chemiluminescence with the ECL kit (Amersham Biosciences). Antibodies sources are as follows: anti-phospho-PKD1-Ser^744/748^, anti-phospho-PKD1-Ser^916^ (also recognize human PKD1-Ser^738/742^ and Ser^910^, respectively), anti-phospho-ERK-Thr^202^/Tyr^204^, anti-PKD1 were obtained from Cell Signaling Technology (Beverly, MA). Anti-phospho-PKD2-Ser^876^ and anti-PKD2 were purchased from R & D Systems (Minneapolis, MN). Anti-PKD3 was obtained from Bethyl Laboratories (Montgomery, TX).

### Measurement of intracellular Ca^2+^ transient by FLIPR®

Jurkat T-cells were serum-starved overnight in the absence or presence of PTX (10 ng/ml) and then washed with Hank’s balanced salt solution (HBSS). Washed cells (1×10^6^ cells/ml) were preloaded with Fluo-4 (AM) followed by incubation at 37°C for 1 h. These labeled cells were then transferred to a black-walled and clear-bottomed 96-well plate (1×10^5^ cells/well) placed in the Fluorometric Imaging Plate Reader (FLIPR), and 50 μl of HBSS (with or without agonists) was added to each well. The resulting fluorescent signals that reflect the intracellular Ca^2+^ transients were monitored by an excitation wavelength of 488 nm and detection with the emission wavelength from 510 to 570 nm.

### Co-immunoprecipitation assay

Transfected cells were lysed in the lysis buffer as described before. Cell lysates were centrifuged (12000 g, 4°C, 5 min) to remove cellular debris. Lysates were incubated at 4°C overnight with M2 affinity gels (20 μl/ sample) for the binding with Flag-tagged Gβ subunits. The resulting immunoprecipitates were collected by centrifugation at 1,000 g, 4°C, for 3 min and then washed three times with 500 μl lysis buffer. Bound proteins were eluted by 50 μl of lysis buffer and 10 μl of 6× SDS-containing sample buffer, and boiled μfor 5 min prior to separation by 12% SDS-polyacrylamide gel electrophoresis (PAGE). Flag-tagged Gβ, HA-tagged Gγ subunits, PLCβ_2_ and PKD1 in the immunoprecipitates were detected by their corresponding antisera followed with horseradish peroxidase-conjugated secondary antisera in Western blotting analysis.

### Chemotactic assay

The chemotactic ability of Jurkat T cells was evaluated using transwell plates (Costar, Cambridge, MA) with polycarbonate inserts with 5-μm pores (Costar 3421). Lower chambers were loaded with 600 μl of migration media alone or containing SDF-1α at the concentration of 100 nM. Cells (0.1 ml) at 1 × 10^6^/ml were added to the top chamber of a 24-well transwell (6.5-μm diameter, 5-μm pore size) and incubated for 4 h at 37°C. The cells which passed through the membranes and migrated to the lower chambers were quantified under microscopy.

### Statistics

The values shown in each figure represent mean ± SEM from at least three individual experiments. Statistical analyses were performed by ANOVA, followed by the Bonferroni’s post test. Differences with a value of P < 0.05 were considered statistically significant.

## Results

Previous studies on Gα subunit-induced activation of PKD isoforms were primarily performed on the PKD1 prototype with Gα_q_[30,31], leaving the activation profile of the PKD family rather incomplete. Most of these studies employed aluminum tetrafluoride (AlF_4_^−^) to elicit G protein-mediated activation of PKD. Although AlF_4_^−^ can selectively stimulate heterotrimeric G proteins over monomeric GTPases [32,33], AlF_4_^−^ activates multiple heterotrimeric G proteins simultaneously and thus cannot be used to identify the specific G proteins involved in the activation of PKD. On the basis of these considerations, we aimed to firstly define the role of different Gα subunits in promoting the activation of all three PKD isoforms. We performed screening on Gα subunit-mediated PKD1 phosphorylation. HEK293 cells were transfected with wild-type (WT) or constitutively active (RC/QL) Gα subunits (Gα_q_, Gα_11_, Gα_14_, Gα_16_, Gα_12_, Gα_13_, Gα_i1_, Gα_i2_, Gα_i3_, Gα_z_ and Gα_s_) and then assayed for PKD phosphorylation by phospho-PKD-specific antibodies. HEK293 cells have previously been shown to express all three PKD isoforms [34].

**Figure 1:**
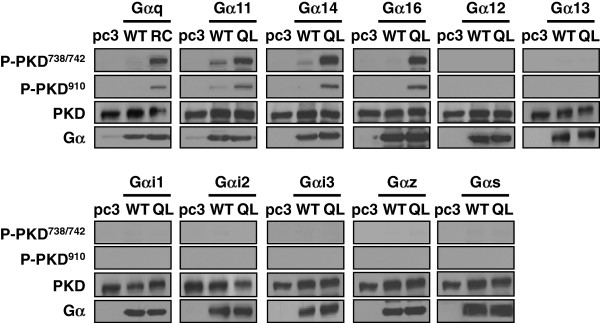
**Constitutively active mutants of G**_**q **_**family members stimulate PKD1 phosphorylation.** HEK293 cells were transfected with pcDNA3, wild type (WT) or constitutively active (RC or QL) Gα subunits of G_q_, G_12_, G_i_ and G_s_ families. Transfectants were then lysed, and proteins were subjected to SDS-PAGE and immunoblotting with antibodies against phospho-PKD1, PKD1, and specific Gα subunits.

The phosphorylation of a pair of highly conserved serine residues in the activation loop (Ser^738^ and Ser^742^ in PKD1; Ser^706^ and Ser^710^ in PKD2; Ser^731^ and Ser^735^ in PKD3) plays a crucial role in human PKD activity [35]. Some early studies on PKD targeted the autophosphorylation sites (Ser^916^ in PKD1 and Ser^876^ in PKD2) as surrogate markers of mouse PKD activity, though a recent report has demonstrated that this site is not required for activation [36]. Therefore, anti-phospho-PKD1 Ser^744/748^ and Ser^916^ antibodies (also recognize human PKD1 at Ser^738/742^ and Ser^910^, respectively) were both adopted for the evaluation of PKD1 activation. As shown in Figure 1, expression of WT Gα subunits did not induce significant PKD1 phosphorylation as compared to the vector control, although expression of Gα_11_ or Gα_14_ slightly enhanced the basal PKD phosphorylation. Conversely, prominent phosphorylation of PKD1 was observed in cells expressing one of the constitutively active mutants from the Gα_q_ subfamily (Gα_q_, Gα_11_, Gα_14_, or Gα_16_). Western blot analysis verified that the expression levels of PKD1 were similar and that both WT and constitutively active Gα subunits were expressed at comparable levels (Figure 1). In contrast, there was no detectable phosphorylation of PKD1 by constitutively active mutants from G_i_, G_s_, or G_12_ subfamilies (Figure 1). This is consistent with earlier studies demonstrating that the constitutively active mutants of Gα_12_ and Gα_13_ did not induce PKD activation in COS-7 cells [30].

**Figure 2:**
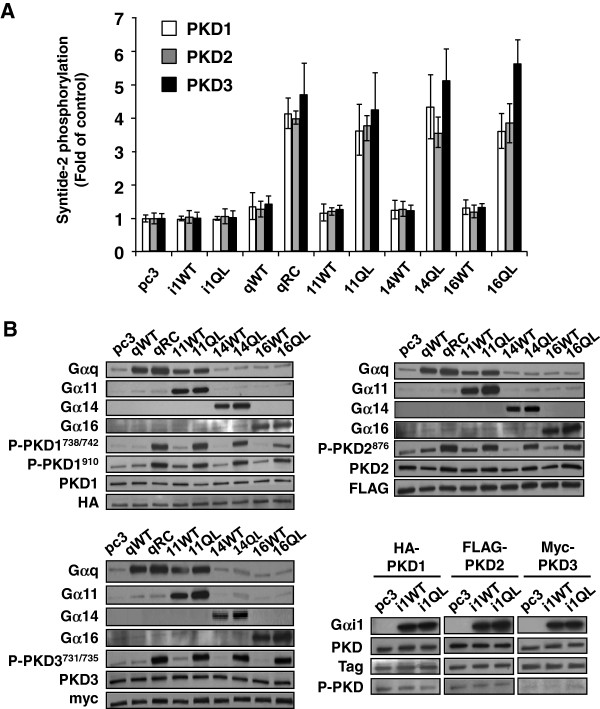
**All Gα subunits from the G**_**q **_**subfamily can activate PKD1, PKD2 and PKD3.** (**A**) Stably expressed and epitope-tagged PKD isoforms were immunoprecipitated from HEK293 cells transiently co-transfected with Gα subunits, and syntide-2 phosphorylation assays were carried out as described under “Materials and Methods”. Results are the average of at least three independent experiments, and presented as fold of control (±S.E.M.). (**B**) Cell lysates from HEK293 transfectants as described in (**A**) were subject to Western blot analysis using antibodies against specific Gα subunits (except anti-Gα_q_ which cross reacts with Gα_11_), phosphorylated PKD isoforms, PKD1, 2, 3 as well as their corresponding tag. Similar results were obtained in three separate experiments.

To examine whether Gα subunits from the G_q_ subfamily are all capable of inducing activation of all three isoforms of PKD, HEK293/HA-PKD1, HEK293/FLAG-PKD2 and HEK293/Myc-PKD3 stable cell lines were established and then transiently transfected with WT or the RC/QL mutants of Gα subunits (Gα_q_, Gα_11_, Gα_14_, Gα_16_), followed by *in vitro* [^32^P]-kinase assays using syntide-2 as an exogenous substrate for PKD. As shown in Figure 2A, PKD isoforms isolated from all three stable cell lines transfected with vector control or plasmids encoding the WT Gα subunits exhibited low catalytic activity. In contrast, those immunoprecipitated from stable cell lines overexpressing a constitutively active mutant displayed marked increase in PKD kinase activity. Comparable expressions of Gα subunits and PKD isoforms in the various transfectants were confirmed by Western blot analyses (Figure 2B). We also examined the phosphorylation of specific PKD isoforms in the same samples. Since anti-phospho-PKD1^738/742^ exhibits some cross-reactivity with PKD2 and PKD3, anti-phospho-PKD1^910^ was also employed to detect PKD1 phosphorylation. Likewise, anti-phospho-PKD2^876^ was used for PKD2. As PKD3 lacks the phosphorylation site equivalent to phospho-PKD1^910^, only the phosphorylation at PKD3^731/735^ was monitored. In agreement with the results from the *in vitro* kinase assay, stimulatory PKD phosphorylation for all three PKD isoforms was enhanced in the presence of constitutively active Gα mutants from the G_q_ subfamily (Figure 2B). Unlike members of the G_q_ subfamily, constitutively active Gα_i1_ failed to stimulate the kinase activity of all three forms of PKD (Figure 2A) or elevate their level of phosphorylation (Figure 2B). Similar results were obtained with other members of the G_i_ (Gα_i2_, Gα_i3_, Gα_oA_, Gα_z_, Gα_t1_, and Gα_t2_), G_s_ (Gα_sL_ and Gα_olf_) and G_12_ (G_12_ and G_13_) families (Additional file 1: Figure S1 and Additional file 2: Figure S2). Collectively, these results demonstrated that PKD1, PKD2 and PKD3 can be specifically activated by the constitutively active Gα subunits from the G_q_ family, but not by those of G_i_, G_s_ or G_12_ families.

**Figure 3:**
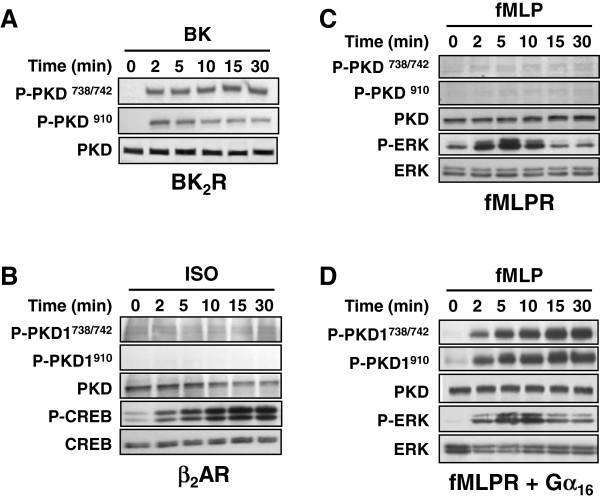
**G**_**q**_**, but not G**_**s **_**or G**_**i **_**signaling is linked to stimulatory phosphorylation of PKD in HEK293 cells.** HEK293 cells were stably transfected with BK_2_R (**A**), β_2_AR (**B**) or fMLPR, in the absence (**C**) or presence of Gα_16_ (**D**). Transfectants were serum starved for 4 h prior to treatment with 100 nM bradykinin (BK), 10 μM isoproterenol (ISO) or 300 nM N-formyl-methionyl-leucyl-phenylalanine (fMLP) for the indicated durations. Cell lysates were resolved in SDS-PAGE, and the presence of ERK, PKD and CREB phosphorylation was detected by their respective anti-phospho or anti-total antisera. Activation of PKD was observed only for BK_2_R and fMLPR/Gα_16_ stable cells. CREB activation served as a positive control for β_2_AR stable cells.

The preceding experiments suggest that the Gα subunits from the G_q_ family contribute to elevated PKD phosphorylation. To examine in more detail the stimulation of PKD by G protein signaling, we tested different G_q_-, G_s_- and G_i_-coupled receptors for their ability to activate PKD1 in HEK 293 cells. HEK293 cells were transfected with the G_q_-coupled bradykinin BK_2_ receptor (Figure 3A), G_s_-coupled β_2_-adrenergic receptor (Figure 3B) or G_i_-coupled fMLP receptor (Figure 3C), and the transfectants subsequently examined for agonist-induced PKD1 activation. Phosphorylation of CREB or ERK was simultaneously monitored as positive controls of G_s_- and G_i_-signaling, respectively. In line with the data in Figures 1 and 2, only bradykinin (which stimulates the G_q_-coupled BK_2_ receptor) rapidly and potently stimulated PKD1 phosphorylation (Figure 3A), while isoproterenol and fMLP failed to induce any detectable PKD activation despite obvious phosphorylation of CREB or ERK (Figure 3B and C). Since many G_i_-coupled receptors including the fMLP receptor are capable of interacting with Gα_16_[37], it is expected that co-expression of Gα_16_ would turn on G_q_-related signals, thus allowing effective stimulation of PKD1 phosphorylation. As illustrated in Figure 3D, prominent fMLP-induced PKD1 phosphorylations at both Ser^738/742^ and Ser^910^ were observed in HEK293 cells co-expressing the G_i_-coupled fMLP receptor and Gα_16_ (Figure 3D); the fMLP-induced response was readily detected by 2 min and was maintained up to 30 min. These results further confirmed the specificity of Gα_q_-mediated PKD activation and implied that many GPCRs are capable of regulating the function of PKD through members of the G_q_ subfamily. This may have particular relevance to hematopoietic cells since the promiscuous Gα_16_ and Gα_14_ are mainly expressed in immune cells and are capable of recognizing a large number of GPCRs [38,39].

**Figure 4:**
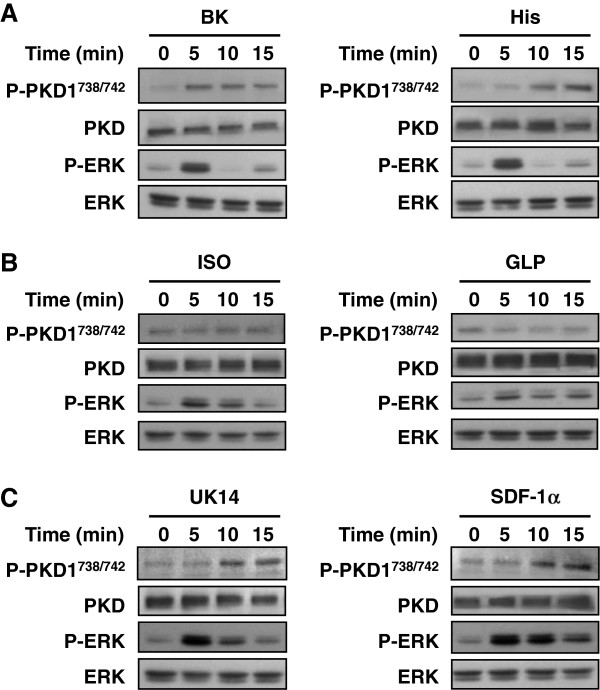
**Stimulation of Gq/Gi-coupled receptors activates PKD1 in HeLa cells.** HeLa cells were serum starved, followed by treatment with (**A**) 100 nM bradykinin (BK) or 10 μM histamine (His); (**B**) 10 μM isoproterenol (ISO) or 100 nM glucagon-like peptide (GLP); (**C**) 10 μM UK14304 (UK14) or 100 nM stromal cell-derived factor-1 (SDF-1) for the indicated times. Cell lysates were detected for phospho-PKD, PKD, phospho-ERK and ERK with their respective anti-sera.

Next, we investigated whether PKD phosphorylation can be induced upon activation of G_q_-coupled receptors that are endogenously expressed in HeLa cells. Serum starved HeLa cells were treated with various agonists targeting G_q_-, G_i_- and G_s_-coupled receptors for various durations, and PKD1 phosphorylation was determined by Western blot analysis. As expected, bradykinin and histamine acting on G_q_-coupled receptors effectively induced a marked increase in PKD phosphorylation at the activation loop (Figure 4A). Agonists that act on G_s_-coupled β-adrenergic receptor (isoproterenol) and GLP receptor (glucagon-like peptide) failed to activate PKD, even when stimulatory phosphorylation of ERK was clearly detected (Figure 4B). Unexpectedly, stimulation of G_i_-coupled α_2_-adrenergic receptor (by UK14304) and CXCR_4_ receptor (by SDF-1α) led to observable PKD activation. This is in contrast to the result presented in Figure 3C where stimulation of the G_i_-coupled fMLP receptor in HEK293 cells failed to promote PKD activation.

**Figure 5:**
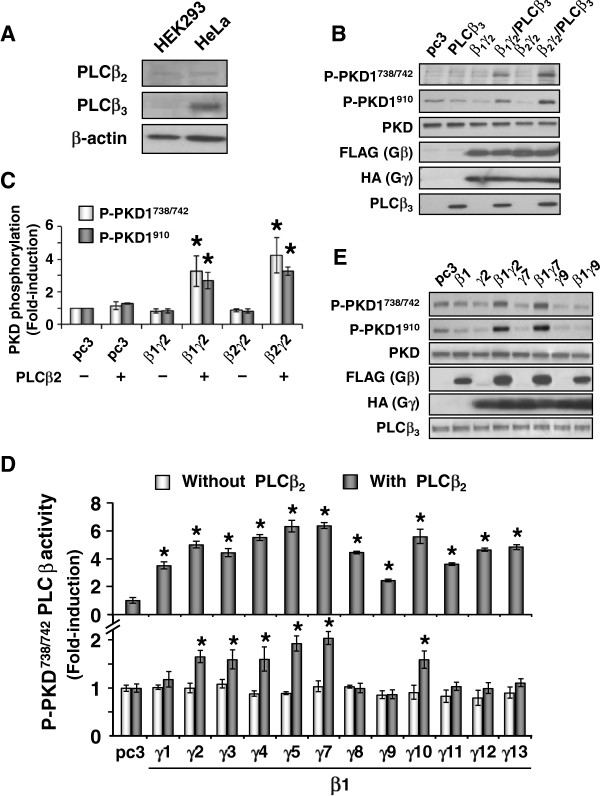
**PLCβ**_**2/3 **_**is required for Gβγ dimer-induced PKD activation.** (**A**), Expression of PLCβ_2_ and PLCβ_3_ in HEK293 and HeLa cells was determined with their respective anti-sera. HEK293 cells were transfected with pcDNA3 and Gβγ dimers with or without PLCβ_3_ (**B**) or PLCβ_2_ (**C**). PKD activation was detected by specific phospho-antibodies. Results shown are the mean ± S.E.M. of at least three independent experiments. (**D**) HEK293 cells were transfected with pcDNA3, PLCβ_2_, Gβγ with or without PLCβ_2_. Transfectants were lysed and the extracts analyzed by PLC assay or SDS-PAGE together with Western blot using antibodies against phosphorylated PKD1. Band intensity was quantified by Image J software (National Institute of Health, Bethesda, MD, USA) and depicted in graphical form, and presented as a fold-induction of the pcDNA3 control. Results shown are the mean ± S.E.M. of at least three independent experiments. (**E**) HeLa cells were transiently transfected with HA-tagged PKD1 together with vector control or Gβγ dimers. The expression of PKD1, FLAG-Gβ, HA-Gγ and endogenous PLCβ_3_ were detected with their specific antibodies.

The ability of G_i_-coupled receptors to stimulate PKD phosphorylation in HeLa cells was contrary to the results obtained with either Gα_i_QL (Figures 1 and 2) or the G_i_-coupled fMLP receptor in HEK293 cells (Figure 3C). Given that Gα_q_-induced activation of PKD is known to be mediated via PLCβ/PKC [30], and that Gα_i_ apparently could not activate PKD, we hypothesized that PKD activation by the G_i_-coupled receptors in HeLa cells was mediated by the Gβγ subunits, presumably via Gβγ-sensitive PLCβ_2_ or PLCβ_3_. Gβγ-induced activation of PKD in HeLa cells have indeed been reported [27]. To test this hypothesis, we first examined the endogenous expression of PLCβ_2_ and PLCβ_3_ in both HEK293 and HeLa cells. Western blot analysis revealed that HEK293 cells expressed barely detectable levels of PLCβ_2_ and PLCβ_3_, whereas PLCβ_3_ (but not PLCβ_2_) was abundantly expressed in HeLa cells (Figure 5A).

To determine the importance of Gβγ-sensitive PLCβ_2/3_ in Gβγ-mediated PKD activation, HEK293/Gγ_2_ stable cells were transiently transfected with FLAG-Gβ_1–2_, in the absence or presence of PLCβ_2/3_. Because consistent expression of Gγ subunits (~6 kDa) is more difficult to achieve in transient transfections, HEK293 cells stably expressing Gγ_2_ were employed in these assays. As expected, co-expression of various combinations of Gβγ alone did not induce any stimulatory phosphorylation as compared to the vector control in HEK293 cells (Additional file 3: Figure S3A). Upon co-expression with PLCβ_3_, however, both Gβ_1_γ_2_ and Gβ_2_γ_2_ markedly enhanced the level of PKD phosphorylation; the expression of PLCβ_3_ alone had no significant effect on PKD phosphorylation (Figure 5B). Likewise, co-expression of Gβ_1_γ_2_ or Gβ_2_γ_2_ with PLCβ_2_ induced significant PKD phosphorylation (Figure 5C). These results not only suggest the crucial role of PLCβ_2/3_ in Gβγ-mediated PKD activation, but also help to explain the differences in G_i_-mediated PKD phosphorylation in HEK293 and HeLa cells.

Since the Gγ subunit identity has been shown to affect signaling specificity [24-26], we determined whether other Gβ_1_γ dimer combinations can effectively induce PKD1 activity in the presence of PLCβ_2/3_. Hence, HEK293 cells were transfected with pcDNA3 (vector control) and one of the twelve combinations of Gβ_1_γ_x_ dimer, with or without PLCβ_2_. As shown in Figure 5D (lower panel), transfection of Gβγ dimers alone did not significantly enhance the phosphorylation of PKD1 beyond the vector control. Among all of the Gβ_1_γ_x_ combinations tested, Gβ_1_γ_2_, Gβ_1_γ_3,_ Gβ_1_γ_4,_ Gβ_1_γ_5,_ Gβ_1_γ_7_ and Gβ_1_γ_10_ consistently triggered strong and significant PKD1 phosphorylation upon co-expression with PLCβ_2_, however, there was no significant change in PKD1 phosphorylation in other Gβ_1_γ_x_/PLCβ_2_-overexpressing cells (Figure 5D, lower panel). Comparable expressions of all Gβ_1_γ_x_ combinations and PLCβ_2_ were detected in the transfectants (data not shown), resulting in elevated levels of IP_3_ formation (Figure 5D, upper panel) as reported previously [21]. We also tested whether selected Gβ_1_γ_x_/PLCβ_2_ combinations can induce *in vitro* kinase activity of the different PKD isoforms (PKD1-3). In agreement with the Gβ_1_γ_x_/PLCβ_2_-induced PKD1 phosphorylation profile, Gβ_1_γ_2_/PLCβ_2_ and Gβ_1_γ_7_/PLCβ_2_ induced significant PKD kinase activity with all three PKD isoforms, while Gβ_1_γ_9_/PLCβ_2_ failed to do so (Additional file 3: Figure S3B). Similar Gβ_1_γ_x_-mediated PKD activation profile was obtained with PLCβ_3_ (data not shown). As expected, Gβ_1_γ_x_ failed to induce PKD phosphorylation with PLCβ_1_ which is insensitive to Gβγ (Additional file 3: Figure S3C).

Having demonstrated that certain Gβ_1_γ_x_/PLCβ_2/3_ combinations were more effective in triggering PKD activity in HEK293 cells, we asked if similar Gβ_1_γ_x_ selectivity for PKD phosphorylation could be observed in HeLa cells that endogenously express high level of Gβγ-sensitive PLCβ_3_ (Figure 5A). Due to the relatively low levels of endogenously expressed PKD1 [34], HeLa cells were transiently co-transfected with cDNAs encoding PKD1 and Gβ_1_γ_2_, Gβ_1_γ_7_ or Gβ_1_γ_9_, followed by serum starvation and subsequent immuno-detection of stimulatory phosphorylated PKD. The results obtained with endogenous PLCβ_3_-expressing HeLa cells (Figure 5E) were essentially similar to those obtained from the PLCβ_2/3_-transfected HEK293 cellular background (Figure 5D, lower panel). This further indicates that the identity of the Gγ subunit may confer specificity to Gβγ-mediated PKD phosphorylation.

**Figure 6:**
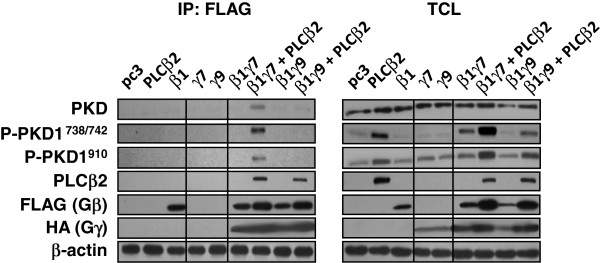
**Gβ**_**1 **_**interacts with PKD1 in the presence of specific Gγ subunits and PLCβ**_**2**_**.** HEK293 cells were transiently transfected with pcDNA3, PLCβ_2_, and different Gβγ combinations as indicated, followed by serum starvation. Cell lysates were subjected to immunoprecipitation by FLAG-tagged affinity gel and SDS-PAGE to resolve for proteins bound to FLAG-Gβ_1_. Black lines represent positions at which images from the same blot but on different lanes were merged.

It has previously been suggested that Gβγ activates PKD through direct interaction at its PH domain [27]. However, overexpression of Gβγ dimers failed to stimulate PKD phosphorylation in HEK293 cells (Figures 5B-D and Additional file 3: Figure S3A-B) unless Gβγ-responsive PLCβ_2/3_ was co-expressed (Figures 5D and Additional file 3: Figure S3B-C). Despite the fact that all of the functional Gβ_1_γ_x_ dimers tested are capable of stimulating PLCβ activity [21], only certain Gβ_1_γ_x_ dimers (e.g. Gβ_1_γ_2_) effectively stimulated PKD phosphorylation in the presence of PLCβ_2/3_ (Figure 5D, lower panel). Hence, we hypothesized that the presence of PLCβ_2/3_ may allow specific Gβγ to associate with PKD. For this, HEK293 cells were transiently transfected with pcDNA3 (vector control), Gβ_1_γ_x_ (Gβ_1_γ_7_, Gβ_1_γ_9_) with or without PLCβ_2_. FLAG-tagged Gβ_1_ was immunoprecipitated from the lysates of the transfectants, and the immune complexes were subjected to SDS-PAGE, followed by Western blotting for any PKD co-immunoprecipitated with Gβ_1_. As shown in Figure 6, phosphorylated PKD1 was clearly detectable in the immunoprecipitates prepared from transfectants expressing both Gβ_1_γ_7_ dimer and PLCβ_2_, but not when PLCβ_2_ was absent. Despite comparable expressions of the various constructs (Figure 6, right panel), hardly any PKD1 was pulled down by the FLAG-tagged Gβ_1_ in cells expressing Gβ_1_γ_9_ with or without PLCβ_2_ (Figure 6, left panel). It should be noted that both Gβ_1_γ_7_ and Gβ_1_γ_9_ were able to interact with PLCβ_2_ in a comparable manner because the latter was detected in the immunoprecipitates (Figure 6, left panel). As the current data showed that Gβγ dimers alone are ineffective in the co-immunoprecipitation with PKD, hence, our findings not only demonstrate the crucial role of PLCβ_2_ for the effective binding between Gβγ dimers and PKD, but also implicate that only specific Gβγ dimers are capable of interacting and activating PKD in the presence of PLCβ_2_.

**Figure 7:**
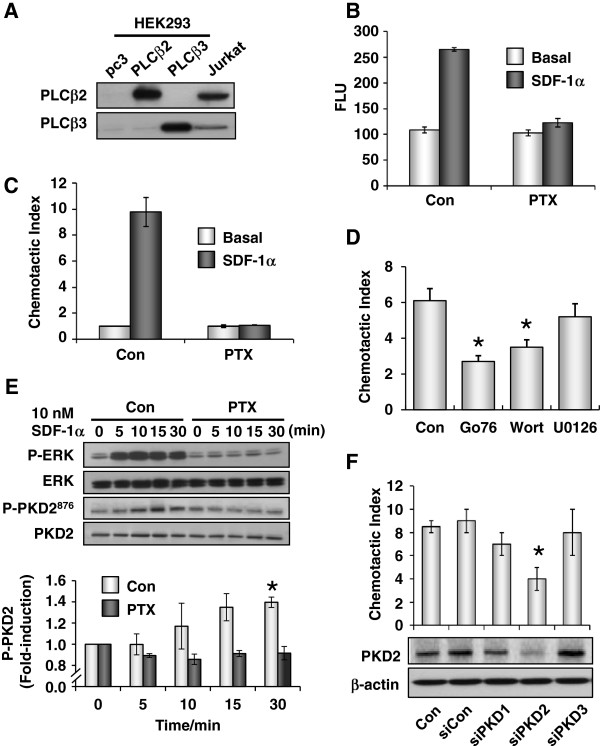
**SDF-1α-induced chemoataxis in Jurkat T cells is dependent on PTX-sensitive G**_**i **_**proteins and PKD2.** (**A**) Expression of PLCβ_2/3_ in Jurkat T cells were examined alongside with parental HEK293 cells or HEK293 cells overexpressing PLCβ_2/3_. (**B**) Bar diagram showing maximum SDF-1α-induced Ca^2+^ mobilization in Jurkat T cells with or without PTX pretreatment. (**C**) Jurkat T cells pretreated with or without PTX were subjected to SDF-1α-induced chemotactic assay. The chemotactic index was expressed as the ratio of the numbers of cells found in the lower compartments, between the agonist-stimulated and the unstimulated groups. (**D**) Jurkat T cells were pretreated with or without specific inhibitors for PKD (100 nM Gö6976), PI3K (100 nM wortmannin), and MEK/ERK (10 μM U0126) and then subjected to the chemotactic assay in the presence of SDF-1α. (**E**) Jurkat T cells pretreated with or without PTX were stimulated with 10 nM SDF-1α for the indicated durations. Cell lysates were subjected to immunoblotting for ERK and PKD2 phosphorylation. (**F**) Jurkat T cells were transfected with vehicle (RNase free water), scrambled siRNA (siCon), siPKD1, siPKD2 or siPKD3 oligonucleotides for 72 h prior to chemotactic assay. Cells were also harvested and lysates were subjected to Western blot analysis with specific antibody against PKD2. β-actin was used as loading control.

Having established that PKD1-3 activation is promoted by ectopic expression of certain Gβγ complexes, we investigated whether Gβγ-mediated PKD activation was implicated in G_i_-linked biological function. Cell migration [34] and invasion [40] represent some of the known cellular functions of PKD. Since Jurkat T-cells express the G_i_-coupled receptor CXCR_4_ and it is responsive to stromal cell-derived factor 1α (SDF-1α) for chemotaxis [41], it appears to be a good cellular system for this investigation. First of all, we examined whether PLCβ_2_ and PLCβ_3_ are endogenously expressed in Jurkat T cells. Indeed, Jurkat T-cells endogenously express both PLCβ_2_ and PLCβ_3_ isoforms, with the former being more abundant (Figure 7A). Next, we used PTX (which ADP-ribosylates G_i_ proteins) to confirm that SDF-1α-induced signaling and chemotaxis in Jurkat T-cells are mediated via G_i_ proteins. Both SDF-1α-induced intracellular Ca^2+^ mobilization (Figure 7B) and chemotaxis (Figure 7C) in Jurkat T-cells were completely abolished upon PTX pretreatment. These results imply that CXCR_4_ utilizes G_i_ proteins to stimulate chemotaxis and PLCβ-mediated Ca^2+^ mobilization in Jurkat T cells. The latter response was presumably mediated by Gβγ dimers released from activated G_i_ proteins [42,43].

To determine whether PKD contributed to SDF-1α-induced chemotaxis in Jurkat T cells, we asked if this chemotactic response can be inhibited by the PKD inhibitor, Gö6976. We were able to demonstrate that SDF-1α-induced chemotaxis could be suppressed by pretreatment with Gö6976 (Figure 7D). In agreement with a previous report [41], the PI3K inhibitor wortmannin (Figure 7D) also inhibited the SDF-1α-stimulated chemotaxis. Next, we assessed if PKD can be activated by the G_i_-coupled CXCR4. Jurkat T-cells were pretreated with or without PTX, followed by SDF-1α stimulation. Since Jurkat T-cells predominantly express PKD2 [44], only PKD2 phosphorylation was determined. SDF-1α stimulated PKD2 phosphorylation became evident within 10 min and peaked at 15 min after agonist addition (Figure 7E). The response was effectively abolished by PTX pretreatment of Jurkat T-cells. As a control, phospho-ERK was similarly monitored; SDF-1α also stimulated ERK phosphorylation in a PTX-sensitive manner (Figure 7E).

**Figure 8:**
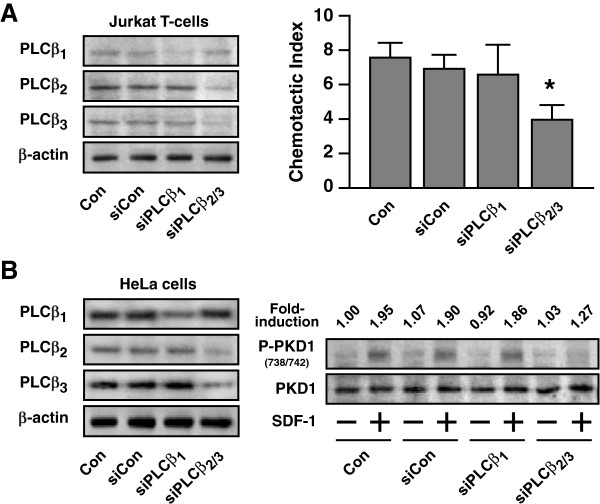
**Requirement of Gβγ-responsive PLCβ**_**2/3 **_**isoforms in SDF-1α-induced signaling activities in Jurkat T-cells and HeLa cells.** (**A**) Jurkat T-cells were transfected with the vehicle control (RNase free water), scrambled siRNA (siCon), siPLCβ_1_, or siPLCβ_2_ and siPLCβ_3_ oligonucleotides for 72 h prior to chemotactic assay upon SDF-1α treatment. Cells lysates were subjected to Western blot analysis with specific antibody against various PLCβ isoforms. β-actin was used as loading control. *Simultaneous knockdown the Gβγ-responsive PLCβ_2_ and PLCβ_3_ isoforms resulted in significant suppression of SDF-1α-induced chemotaxis. (**B**) HeLa cells subjected to similar PLCβ knockdown profiles were examined for SDF-1α-induced PKD activation, the levels of phospho-PKD1 in cell lysates were detected by Western blot analysis. The fold-induction represents the ratio of band intensities (phospho-PKD1) with respect to the basal (1.00) of the control group. Similar results were obtained in two independent experiments.

To substantiate that SDF-1α-induced chemotaxis in Jurkat T-cells is PKD2-dependent, we used specific validated siRNA oligonucleotides to knock down the expression of PKD2. As shown in Figure 7F, control and scrambled siRNAs had no effect on PKD2 expression, while silencing of PKD2 led to a remarkable reduction in PKD2 expression; siRNAs targeting either PKD1 or PKD3 did not affect the expression of PKD2. The siRNA-mediated knockdown of PKD2 effectively inhibited the SDF-1α-induced chemotaxis, whereas the controls and siRNAs targeting PKD1 and PKD3 did not significantly suppress chemotaxis (Figure 7F). Furthermore, silencing of PLCβ_2/3_ but not PLCβ_1_ resulted in the suppression of SDF-1α-induced chemotaxis in Jurkat T-cells, illustrating the importance of Gβγ-responsive PLCβ isoforms in this activity (Figure 8A). As SDF-1α also acts on G_i_-coupled CXCR4 receptor in HeLa cells for PKD activation (Figure 4C), we then performed similar knockdown treatment to verify the possible PLCβ_2/3_-dependency. Our result demonstrated that this G_i_-induced signaling also required the Gβγ-responsive PLCβ_2_/_3_ isoforms to stimulate the PKD activation (Figure 8B).

## Discussion

Extending from prior reports on the regulation of PKD1 by G_q_[30,45], the present study demonstrates unequivocally that each and every member of the G_q_ subfamily (i.e., Gα_q_, Gα_11_, Gα_14_ and Gα_16_) are capable of inducing the kinase activity of all PKD isoforms (Figure 2). The ability to β stimulate PKD activity is apparently unique to the Gα_q_ members because other Gα subunits belonging to the G_i_, G_s_, or G_12_ subfamilies all failed to induce PKD phosphorylation or kinase activity (Figures 1, 2, Additional file 1: Figure S1 and Additional file 2: Figure S2). However, it should be noted that addition of AlF_4_^−^ to cells co-expressing PKD and wild type Gα_13_ can lead to PKD activation [46]. Such an observation is confounded by the fact that AlF_4_^−^ may activate multiple G proteins simultaneously. The lack of effect on PKD by the constitutively active mutant of Gα_13_ has in fact been reported [30]. Hence, it is reasonable to conclude that only members of the G_q_ subfamily are efficiently linked to PKD activation.

Despite the preponderance of G_q_ in mediating GPCR-induced activation of PKD, stimulation of G_i_-coupled receptors in HeLa cells resulted in PKD phosphorylation (Figure 4). This may be explained by the observation that HeLa cells endogenously express Gβγ-responsive PLCβ_2/3_[28], thereby allowing Gβγ released from activated heterotrimeric G_i_ proteins to mediate PKD activation through the Gβγ/PLC/PKC axis. One would expect that stimulation of G_i_-coupled receptors will result in PKD activation in cells endowed with PLCβ_2/3_. However, if the endogenous PLCβ_2/3_ is responsive to Gβγ dimers and all active G protein heterotrimers liberate free Gβγ dimers, then it remains puzzling why stimulation of G_s_-coupled receptors cannot activate PKD via PLCβ_2/3_ (Figure 4B). A recent report has revealed that differential dissociation may exist among different G proteins [47], though it has long been thought that active G protein heterotrimers readily dissociate into Gα*-*GTP subunits and Gβγ dimers [48]. Activated G_oA_ heterotrimers can seemingly dissociate more readily than activated G_s_ heterotrimers, and this may account for Gα-specific activation of Gβγ-sensitive effectors [47]. Alternatively, the lack of G_s_-induced PKD activation may be attributed to insufficient release of Gβγ dimers as most Gβγ-dependent signaling appeared to require substantial amounts of free Gβγ, which is most often achieved by stimulating the more abundantly expressed G_i_ proteins [42,43].

Another interesting observation in the present study pertains to the requirement of PLCβ_2/3_ for Gβγ-induced PKD activation (Figure 5). At first sight, our finding seems to suggest a concept different from the previous belief that Gβγ dimers alone can activate PKD through interaction with the PH domain [27]. However, since the cellular model (i.e. HeLa cells) used in Jamora’s report expresses significant amount of Gβγ-sensitive PLCβ_2/3_, it is possible that the presence of PLCβ_2/3_ enables specific Gβγ dimers to act on the PH domain of PKD. It has been demonstrated that Gγ prenylation is one of the important factors for Gβγ interaction with PLC isoforms, as the presence of farnesyl lipid motif in Gγ_1_, Gγ_9_ and Gγ_11_ may lead to a weaker PLC activation as compared to Gβγ dimers containing other Gγ components with geranylgeranyl lipid motif [49]. Indeed, we have observed that Gβ_1_γ_1_, Gβ_1_γ_9_ and Gβ_1_γ_11_ are associated with a weaker PLC activation and all of them are incapable of effectively stimulating PKD (Figure 5D and 5E). Hence, the possible influence of Gγ prenylation status cannot be neglected. However, Gβ_1_γ_2_ and Gβ_1_γ_3_ induce PLC activities of similar magnitude as those of Gβ_1_γ_12_ and Gβ_1_γ_13_, but only the former two are capable of stimulating PKD. As Gγ_2_, Gγ_3_, Gγ_12_, and Gγ_13_ are commonly incorporated with the geranylgeranyl lipid motif, factors other than Gγ prenylation and PLC activity may also be important for governing the specificity of Gβγ-mediated PKD activation. It can be observed that only certain Gβ_1_γ dimers (i.e., those containing γ_2_, γ_3_, γ_4_, γ_5_, γ_7_, and γ_10_) but not others (i.e., those containing γ_1_, γ_8_, γ_9_, γ_11_, γ_12_, and γ_13_) could effectively activate PKD in the presence of PLCβ_2/3_ (Figure 5D, lower panel). Yet, all combinations of Gβ_1_γ_x_ dimers are capable of activating PLCβ_2_[21]. The differential ability of various Gβ_1_γ dimers to stimulate PKD is thus unlikely to solely depend on their PLCβ activity alone. It can also be observed that the expression levels of Gβ_1_γ_4_, Gβ_1_γ_7_, Gβ_1_γ_9_, Gβ_1_γ_11_ and Gβ_1_γ_12_ appear to be increased upon PLCβ_2_ co-expression (Additional file 4: Figure S4). However, such increased Gβγ expression is not necessarily related to the subsequent PKD activation, as increased Gβ_1_γ_9_, Gβ_1_γ_11_ and Gβ_1_γ_12_ expressions do not effectively stimulate PKD in the presence of PLCβ_2_, whereas Gβ_1_γ_2_, Gβ_1_γ_3_, Gβ_1_γ_5_, and Gβ_1_γ_10_ trigger the kinase activation without increased levels of subunit expressions (Additional file 4: Figure S4). Hence, Gβγ-mediated PKD activation seems to be a specific function in response to unique Gβγ combinations.

In fact, the ability of specific Gβγ dimers to stimulate PKD phosphorylation may depend on their ability to form a complex with PKD, since only those Gβγ dimers that can stimulate PKD (e.g., Gβ_1_γ_7_) could be immunoprecipitated with PKD (Figure 6). The requirement of PLCβ_2/3_ in Gβγ-mediated PKD signaling might be explained if PLCβ_2/3_ is an essential component of the signaling complex that stabilizes the interaction between Gβγ and PKD. The possible existence of a Gβγ/PLCβ_2/3_/PKD signaling complex is supported by the fact that Gβγ dimers serve as direct activators for PLCβ_2/3_[50], probably through the binding of Gβγ to the PH domain of PLCβ_2/3_[51], while Gβγ/PKD-mediated Golgi fragmentation can be inhibited by a sequester peptide with identical sequence of the Gβγ-binding PH domain in PKD [27]. Indeed, we have preliminary data suggesting that PLCβ_2_ can be co-immunoprecipitated with all three PKD isoforms, while PLCβ_1_ fails to do so (Additional file 5: Figure S5). Apparently the reported capabilities of Gβγ to interact with PLCβ_2/3_ and PKD seem to support the notion for the formation of a Gβγ/PLCβ_2/3_/PKD signaling complex. However, it is unclear as to whether a single Gβγ dimer binds to the PH domains of PLCβ_2/3_ and PKD sequentially or simultaneously. Similarly, we cannot rule out the possibility that there may be different pools of Gβγ dimers for Gβγ-PLCβ and Gβγ-PKD interactions, respectively, and that they may subsequently cooperate with each other to stimulate PKD. Further studies are required to examine the precise interactions between Gβγ, PLCβ_2/3_ and PKD.

**Figure 9:**
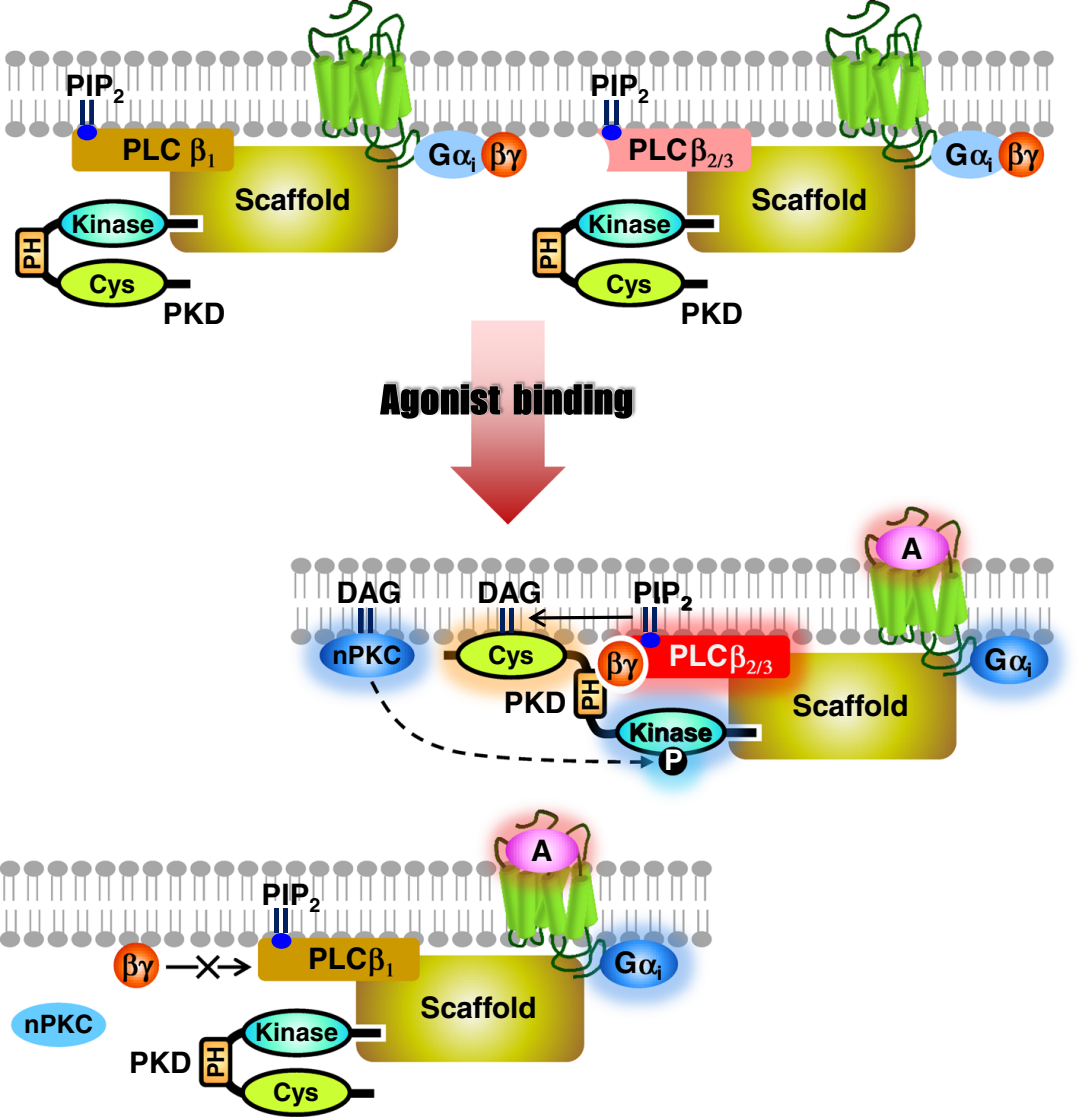
**A schematic diagram for the Gβγ-mediated PKD activation.** PLCβ_2/3_ serves as important intermediates for the Gβγ-mediated PKD activation, in which intracellular scaffold proteins may facilitate or determine the formation of functional complexes among these signaling players. Scaffold proteins (e.g. NHERFs and others) may form functional complexes with specific PLCβ isoforms and PKDs, only those complexes containing PLCβ_2/3_ will enable Gβγ dimers to be recruited and interact with PKDs. The Gβγ/PLCβ_2_/_3_-induced DAG production leads to confirmation changes of PKDs as well as PKC-mediated phosphorylation on the kinases. As demonstrated in the current report, enhanced Gβγ-induced PLCβ_2/3_ stimulation alone does not guarantee a successful PKD activation, it is possible that only specific Gβγ dimers (e.g. Gβ_1_γ_2_) are compatible with the PH domain of PKDs for productive conformation changes, which result in functional activation of PKDs. Such activation mechanism is not feasible for PLCβ_1_ which is Gβγ-insensitive.

The assembly of a Gβγ/PLCβ_2/3_/PKD signaling complex may require the participation of scaffolding proteins. In this regard PKD isoforms have been shown to interact with the PDZ domains of a scaffolding protein family named NHERF [52]. Coincidently, PLCβ_2/3_ can also interact with different NHERF members [53,54]. Hence, NHERF, as well as other similar scaffold proteins, may act as a nexus for Gβγ/PLCβ/PKD signaling (Figure 9), in which intracellular scaffold may facilitate or determine the formation of functional complexes among the signaling players. Scaffolding proteins (e.g. NHERFs and others) may form functional complexes with specific PLCβ isoforms and PKDs, and perhaps only those complexes containing PLCβ_2/3_ will enable Gβγ dimers to be recruited for interaction with PKDs. Such activation mechanism is not feasible for PLCβ_1_ which is Gβγ-insensitive. The Gβγ/PLCβ_2_/_3_-induced DAG production leads to confirmation changes of PKDs as well as PKC-mediated phosphorylation on the kinases. As demonstrated in the current report, enhanced Gβγ-induced PLCβ_2/3_ stimulation alone does not guarantee a successful PKD activation, it is possible that only specific Gβγ dimers (e.g. Gβ_1_γ_2_) are compatible with the PH domain of PKDs for productive conformation changes, which result in functional activation of PKDs. In fact, our unpublished data showed that PKD activation triggered by G_i_-coupled receptors is sensitive to inhibitors for PLCβ (e.g. U73122) as well as to Gβγ subunit scavengers (e.g. transducin). Since only specific Gβγ dimers are capable of stimulating PKD in the presence of PLCβ_2/3_, our results actually suggest a dual requirement of functional PLCβ activity and compatible Gβγ dimers for G_i_-mediated PKD activation. It remains unclear if all the members in the G_q_ family (i.e. Gα_q_, Gα_11_, Gα_14_, and Gα_16_) also activate PKD in a similar manner. However, it should be noted that another scaffold protein named PAR3 have been suggested as a G_q_-specific signaling component with selective recruitment of PLCβ_1_, while PLCβ_2/3_ isoforms may have high preferences towards NHERF members in G_i_-mediated signaling [53,54]. The involvement of different scaffold proteins may also explain the differential observation that, Gα subunits of the G_q_ family (much stronger activators for PLCβ isoforms as compared to Gβγ dimers) are capable of stimulating PKD in a Gβγ-independent manner.

PKD mediates a diverse array of normal biological functions and pathological activities, including cell proliferation and differentiation, cell motility, regulation of cell vesicle trafficking, secretion, and polarity, inflammatory responses, cardiac hypertrophy and cancer [55]. Therein, the transport of protein from the Golgi to plasma membrane is regulated via Gβγ signaling [27,28,56]. From our results, it is postulated that stimulation of G_i_-coupled receptor leads to the liberation of free Gβγ dimers, which then interact with PLCβ_2_/_3_ and activate PKD. This may help to elucidate part of the mechanism regarding secretory activities regulated by receptor-induced Gβγ translocation between the Golgi and plasma membrane [57], and the characteristic of Golgi as one of the major cellular locations for activated PKD [58]. Indeed, Gβγ dimers are known to mediate many cellular responses and signaling pathways involved in multiple aspects of cellular function. Previous studies have reported that SDF-1α-induced activation of CXCR4 receptor induces chemotaxis in Jurkat T cells [41]. Here, our results showed that this G_i_-coupled chemotactic response may be mediated by the Gβγ/PLCβ/PKD axis (Figure 7). However, further investigations are needed to determine whether these components act in concert. The activation of STAT3, which is an important transcription factor, is also regulated by Gβγ-mediated signaling [23]. Similar to PKD, only distinct combinations of Gβγ can effectively activate STAT3. Nevertheless, the panel of STAT3-activating Gβγ dimers is not identical to the PKD-stimulatory Gβγ complexes; only Gβ_1_γ_4_ and Gβ_1_γβ_7_ are effective activators for both pathways. Taken together, our results suggested that PKD may be implicated in diverse cellular activities, including those mediated by Gβγ.

Functional redundancy is a common feature among isoforms of biological molecules. However, it is not always the case. Though the three PKD isoforms are highly conserved and our results showed that all three PKD isoforms (PKD1, PKD2 and PKD3) are activated equally well by Gα subunits from the G_q_ family, as well as by specific Gβ_1_γ_x_ with PLCβ_2/3_, they may have unique functions. For example, PKD1 plays a non-redundant role in pathological cardiac remodeling, and the homozygous germline deletion of PKD1 causes embryonic lethality [59]. As for PKD2, it has a unique role in endothelial cells [6], lymphoid cells [7], and monocytes [34]. Recent studies have revealed the essential role of PKD3 in the progression of prostate cancer [60] and insulin-independent basal glucose uptake in L6 skeletal muscle cells [61]. Further studies are necessary to elucidate the mechanisms behind GPCR-mediated activation of the three PKD isoforms.

## Conclusion

Collectively, among various members of G proteins, only the Gα subunits of the G_q_ family effectively activate all three PKD isoforms (PKD1/2/3), while Gα subunits of other G protein families (G_s_, G_i_, and G_12_) are inefficient in these kinase activations. However, receptors linked to G_i_ proteins are capable of triggering PKD activation in cell lines endogenously expressing (HeLa cells and Jurkat T-cells) or exogenously transfected with (HEK293 cells) Gβγ-sensitive PLCβ_2/3_ isoforms, indicating the involvement of Gβγ dimers for the G_i_-mediated PKD activation. Although the presence of PLCβ_2/3_ is highly important, only those Gβ_1_γ dimers with γ_2_, γ_3_, γ_4_, γ_5_, γ_7_, and γ_10_ are effective activators of PKD, and the specific interaction between Gβγ, PKD and PLCβ_2/3_ may play a pivotal role in this Gβγ-mediated PKD signaling pathway. Furthermore, the biological significance of G_i_-mediated PKD activation is illustrated by SDF-1α-induced chemotaxis on Jurkat T-cells, in which the chemotaxic activity is abolished by pretreatment with PTX and knockdown of PKD. Taken together, our current report illustrates that Gβγ dimers from G_i_ proteins may activate PKD in a PLCβ_2/3_-dependent manner, and the identity of Gγ of the Gβγ dimer being a determinant.

## Abbreviations

BK2R: Bradykinin type II receptor; β2AR: β_2_-adrenergic receptor; DAG: Diacylglycerol; fMLPR: N-formyl-methionyl-leucyl-phenylalanine receptor; GPCR: G protein-coupled receptor; PLCβ: Phospholipase Cβ; PKD: Protein kinase D; PTX: Pertussis toxin.

## Competing interests

The authors declare that they have no competing interests.

## Authors’ contributions

WWIL and ASLC carried out the experiments participated in the design of the study and wrote the manuscript. LSWP and JZ carried out some of the experiments. YHW participated in the design of the study and revised the manuscript. All authors read and approved the final manuscript.

## Supplementary Material

Additional file 1: Figure S1 Constitutively active Gα subunits from the G_i_ subfamily failed to induce PKD activation. (A) HEK293 cells were transiently transfected with pcDNA3 or WT/QL forms of Gα subunits from the G_i_ subfamily. Cell lysates were subjected to SDS-PAGE. Gα subunits, phospho-PKD1^738/742^, phospho-PKD1^910^, total PKD1, tag of PKD1 (HA) were analyzed by Western blotting using respective specific antibody. (B) HA-PKD1, FLAG-PKD2 and Myc-PKD3 were immunoprecipitated from cell lysates described in (A), and subjected to *in vitro* PKD kinase assays in terms of syntide-2 phosphorylation. Results are the average of at least three independent experiments, and represented as fold increase over pcDNA3 control (±S.E.M.). Click here for file

Additional file 2: Figure S2 Constitutively active Gα subunits from the G_s_ and G_12_ subfamilies failed to induce PKD activation. (A) HEK293 cells were transiently transfected with pcDNA3 or WT/QL forms of Gα subunits from the G_s_ and G_12_ subfamilies. Cell lysates were subjected to SDS-PAGE. Gα subunits, phospho-PKD1^738/742^, phospho-PKD1^910^, total PKD1, tag of PKD1 (HA) were analyzed by Western blotting using respective specific antibody. (B) HA-PKD1, FLAG-PKD2 and Myc-PKD3 were immunoprecipitated from cell lysates described in (A), and subjected to *in vitro* PKD kinase assays. Results are the average of at least three independent experiments, and represented as fold increase over pcDNA3 control (±S.E.M.). Click here for file

Additional file 3: Figure S3 PLCβ_2_ and specific Gγ subunits are required in Gβγ-induced PKD activation in HEK293 cells. (A) HEK293 cells stably transfected with pcDNA3 or HA-Gγ_2_ were transfected with pcDNA3, FLAG-Gβ_1_ or FLAG-Gβ_2_. Cell lysates were subjected to SDS-PAGE. FLAG-Gβ, HA-Gγ, phospho-PKD1^738/742^, phospho-PKD1^910^ and total PKD1 were analyzed by Western blotting using respective specific antibody. (B) HEK293 cells were transiently transfected with vector control, PLCβ_2_, various Gβγ dimers and tagged PKD isoforms (HA-PKD1, FLAG-PKD2 and Myc-PKD3). The cultures were then lysed, and the tagged PKD isoforms were immunoprecipitated for *in vitro* PKD kinase assay. (C) HEK293 cells transiently transfected with pcDNA3, Gβ, Gγ_x_, Gβγ combinations with or without PLCβ_1_ or PLCβ_2_ were lysed, and analyzed by Western blotting for PKD1 phosphorylation. Click here for file

Additional file 4: Figure S4 The expression profiles of Gβγ dimers and the corresponding PKD activation in the presence of PLCβ_2_. HEK293 cells were transfected with pcDNA3, PLCβ_2_, various combinations of Gβγ with or without PLCβ_2_. Transfectants were lysed, and the lysates were subjected to Western blotting using antibodies against phosphorylated PKD1, PKD, PLCβ_2_, Flag-tagged Gβ_1_ and HA-tagged Gγ subunits. Click here for file

Additional file 5: Figure S5 PLCβ_2_, but not PLCβ_1_, can be co-immunoprecipitated with various PKD isoforms. HEK293 cells were transiently transfected with pcDNA3, PLCβ_1/2_ with tagged PKD1/2/3 as indicated. HA-PKD1, FLAG-PKD2 and Myc-PKD3 were immunoprecipitated from cell lysates with their respective affinity gels and further analyzed by Western blotting for the possible interaction with PLCβ_1/2_. Click here for file
