# Supplementary figures and images for: Gβγ-mediated activation of protein kinase D exhibits subunit specificity and requires Gβγ-responsive phospholipase Cβ isoforms
Source: Cell Commun Signal. 2013 Apr 5;11:22. doi: 10.1186/1478-811X-11-22 (PMC3637504; doi:10.1186/1478-811X-11-22)

Supplementary Figure (S1)

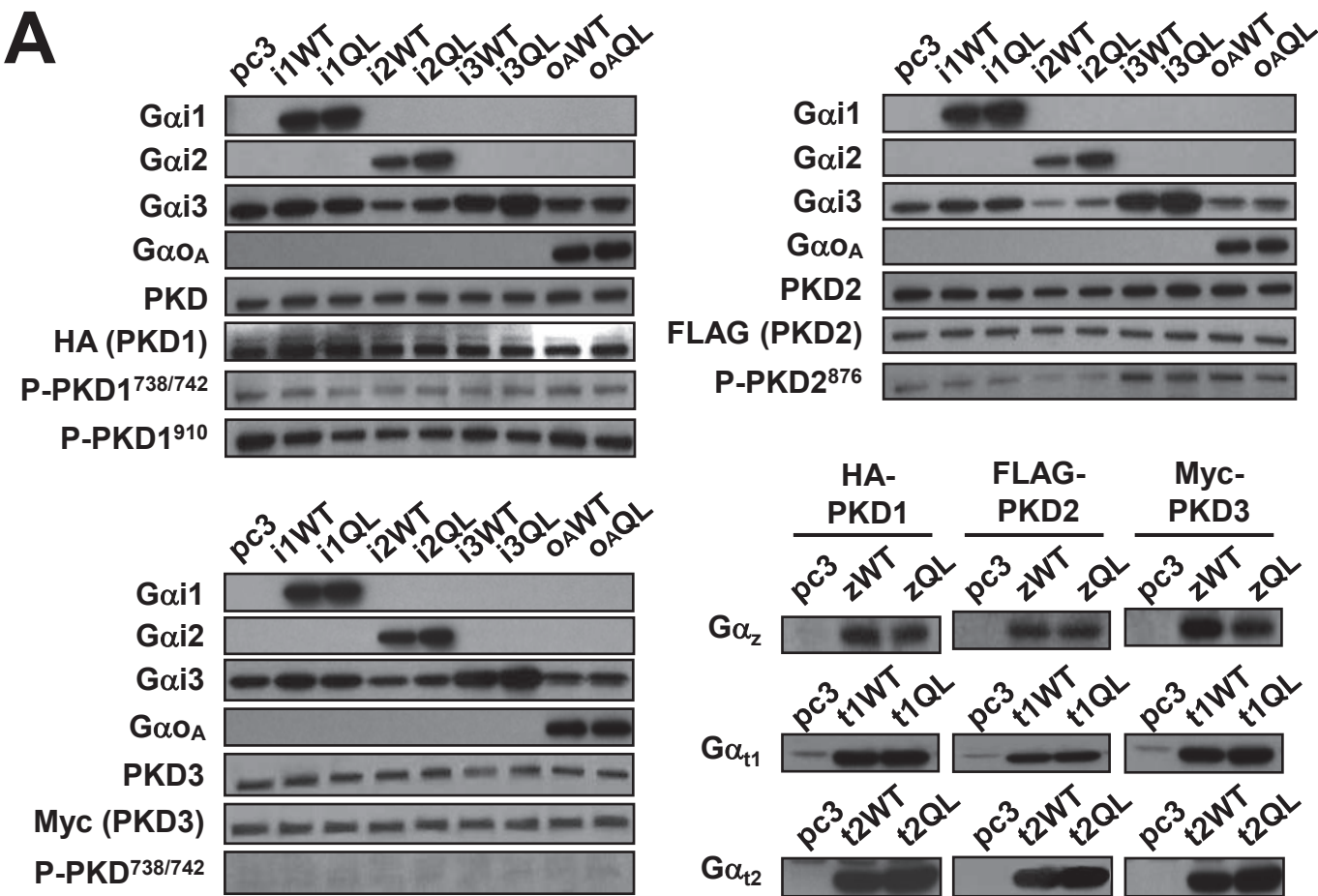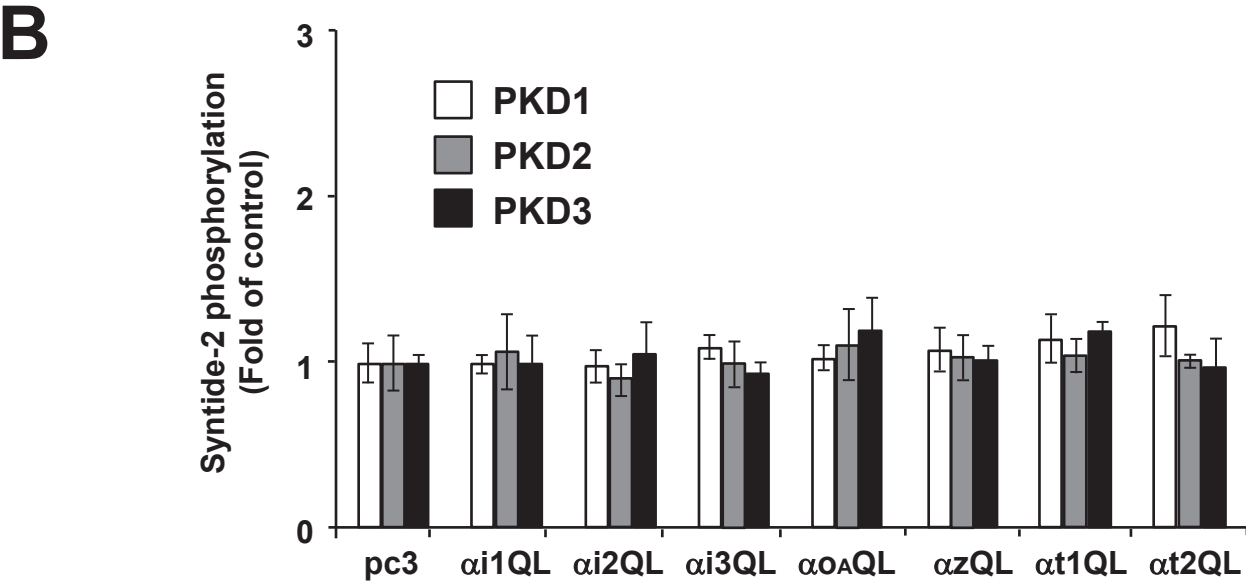

Supplement: Additional file 1: Figure S1 — Constitutively active Gα subunits from the Gi subfamily failed to induce PKD activation. (A) HEK293 cells were transiently transfected with pcDNA3 or WT/QL forms of Gα subunits from the Gi subfamily. Cell lysates were subjected to SDS-PAGE. Gα subunits, phospho-PKD1738/742, phospho-PKD1910, total PKD1, tag of PKD1 (HA) were analyzed by Western blotting using respective specific antibody. (B) HA-PKD1, FLAG-PKD2 and Myc-PKD3 were immunoprecipitated from cell lysates described in (A), and subjected to in vitro PKD kinase assays in terms of syntide-2 phosphorylation. Results are the average of at least three independent experiments, and represented as fold increase over pcDNA3 control (±S.E.M.). [file 1478-811X-11-22-S1.pdf]

# Supplementary Figure (S2)

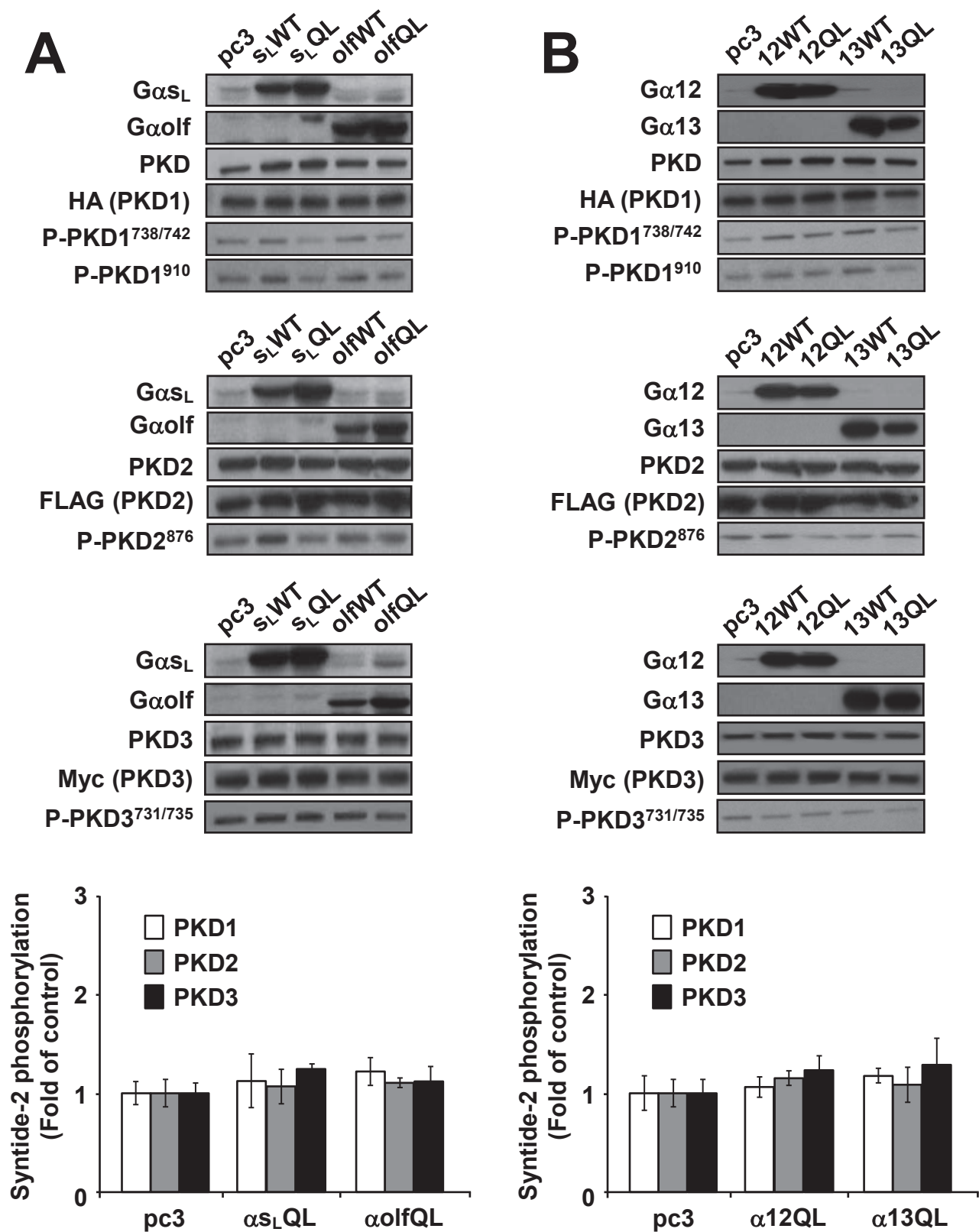

Supplement: Additional file 2: Figure S2 — Constitutively active Gα subunits from the Gs and G12 subfamilies failed to induce PKD activation. (A) HEK293 cells were transiently transfected with pcDNA3 or WT/QL forms of Gα subunits from the Gs and G12 subfamilies. Cell lysates were subjected to SDS-PAGE. Gα subunits, phospho-PKD1738/742, phospho-PKD1910, total PKD1, tag of PKD1 (HA) were analyzed by Western blotting using respective specific antibody. (B) HA-PKD1, FLAG-PKD2 and Myc-PKD3 were immunoprecipitated from cell lysates described in (A), and subjected to in vitro PKD kinase assays. Results are the average of at least three independent experiments, and represented as fold increase over pcDNA3 control (±S.E.M.). [file 1478-811X-11-22-S2.pdf]

Supplementary Figure (S3)

A

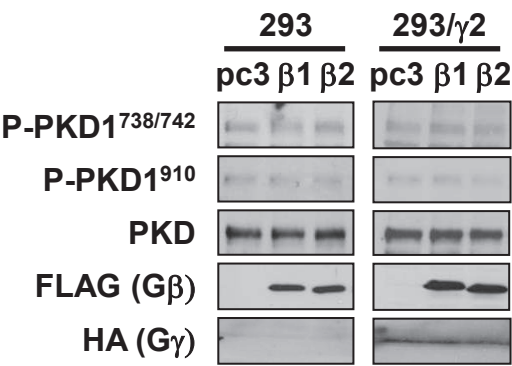

B

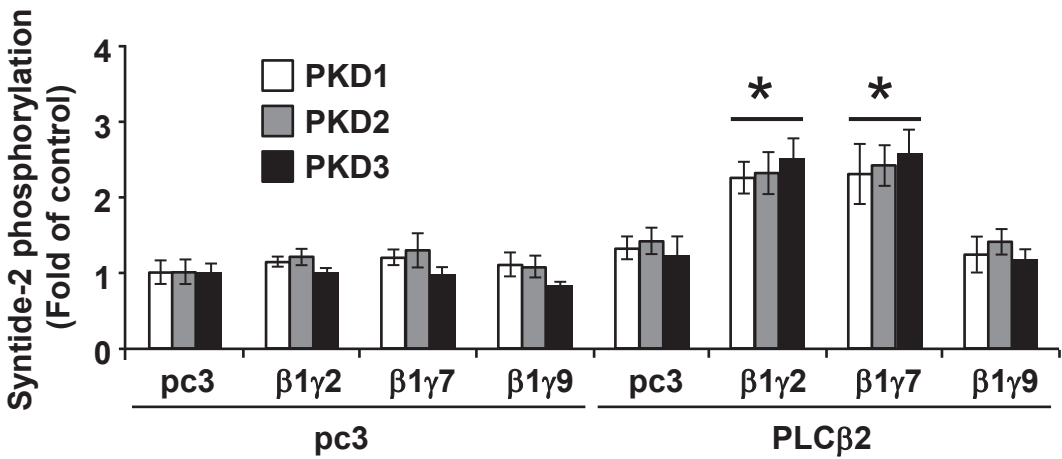

C

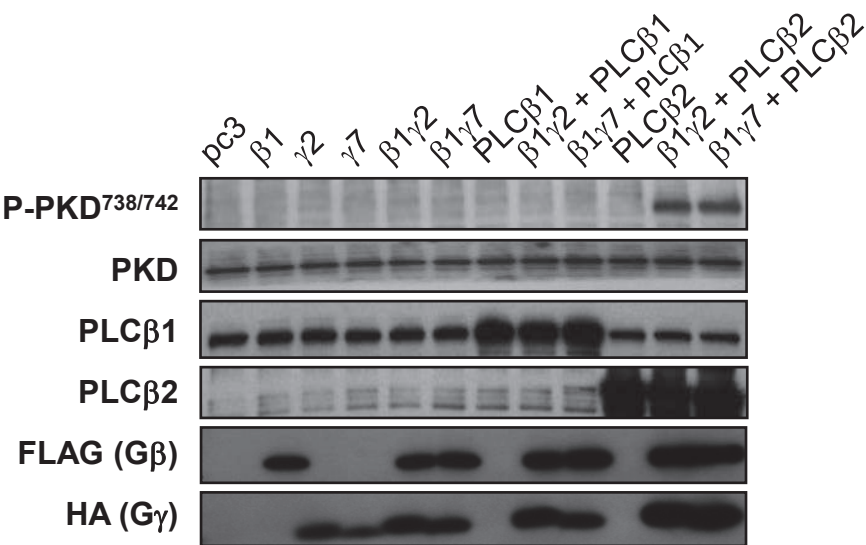

Supplement: Additional file 3: Figure S3 — PLCβ2 and specific Gγ subunits are required in Gβγ-induced PKD activation in HEK293 cells. (A) HEK293 cells stably transfected with pcDNA3 or HA-Gγ2 were transfected with pcDNA3, FLAG-Gβ1 or FLAG-Gβ2. Cell lysates were subjected to SDS-PAGE. FLAG-Gβ, HA-Gγ, phospho-PKD1738/742, phospho-PKD1910 and total PKD1 were analyzed by Western blotting using respective specific antibody. (B) HEK293 cells were transiently transfected with vector control, PLCβ2, various Gβγ dimers and tagged PKD isoforms (HA-PKD1, FLAG-PKD2 and Myc-PKD3). The cultures were then lysed, and the tagged PKD isoforms were immunoprecipitated for in vitro PKD kinase assay. (C) HEK293 cells transiently transfected with pcDNA3, Gβ, Gγx, Gβγ combinations with or without PLCβ1 or PLCβ2 were lysed, and analyzed by Western blotting for PKD1 phosphorylation. [file 1478-811X-11-22-S3.pdf]

Supplementary Figure (S4)

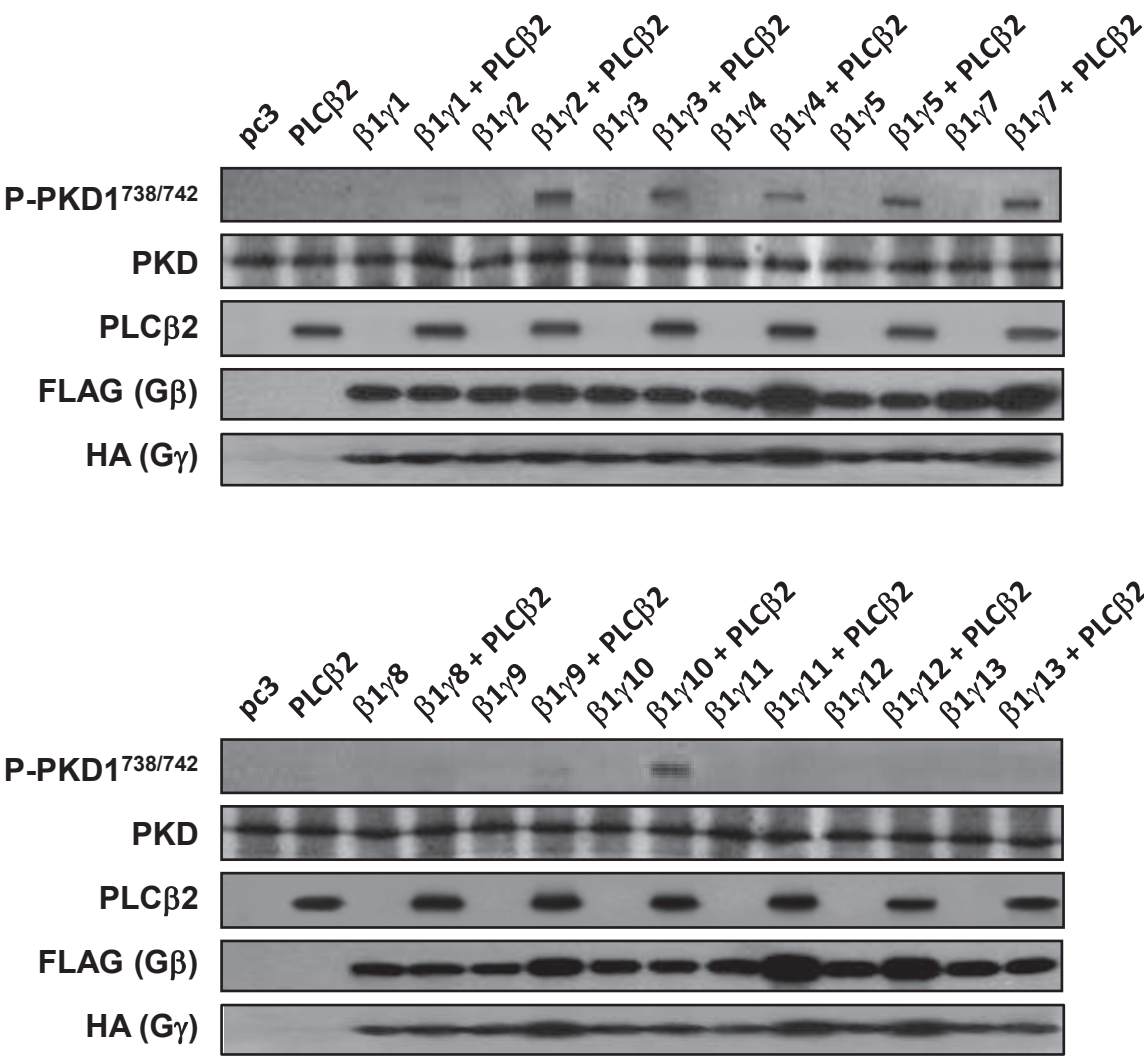

Supplement: Additional file 4: Figure S4 — The expression profiles of Gβγ dimers and the corresponding PKD activation in the presence of PLCβ2. HEK293 cells were transfected with pcDNA3, PLCβ2, various combinations of Gβγ with or without PLCβ2. Transfectants were lysed, and the lysates were subjected to Western blotting using antibodies against phosphorylated PKD1, PKD, PLCβ2, Flag-tagged Gβ1 and HA-tagged Gγ subunits. [file 1478-811X-11-22-S4.pdf]

Supplementary Figure (S5)

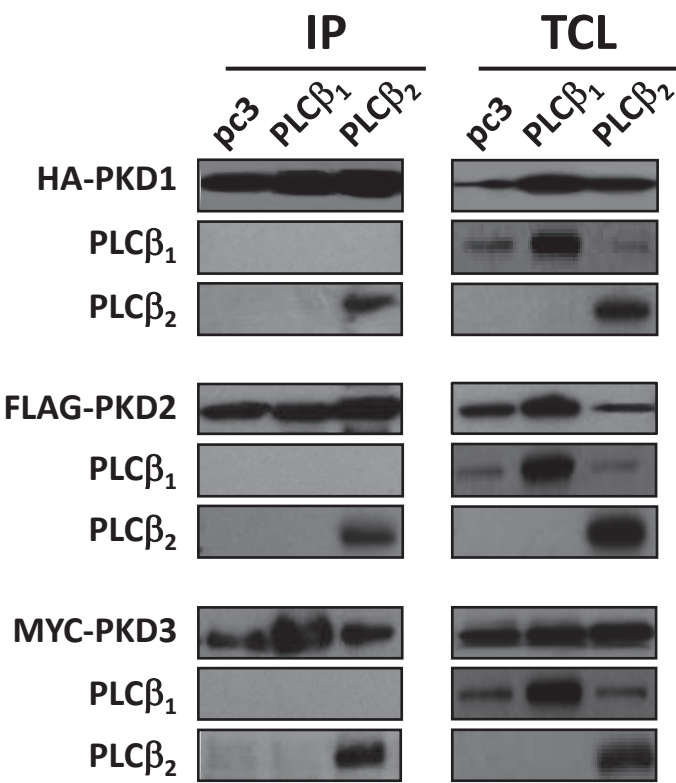

Supplement: Additional file 5: Figure S5 — PLCβ2, but not PLCβ1, can be co-immunoprecipitated with various PKD isoforms. HEK293 cells were transiently transfected with pcDNA3, PLCβ1/2 with tagged PKD1/2/3 as indicated. HA-PKD1, FLAG-PKD2 and Myc-PKD3 were immunoprecipitated from cell lysates with their respective affinity gels and further analyzed by Western blotting for the possible interaction with PLCβ1/2. [file 1478-811X-11-22-S5.pdf]
